# Longitudinal trends in organophosphate incidents reported to the National Pesticide Information Center, 1995–2007

**DOI:** 10.1186/1476-069X-8-18

**Published:** 2009-04-20

**Authors:** David L Stone, Daniel L Sudakin, Jeffrey J Jenkins

**Affiliations:** 1 National Pesticide Information Center, Department of Environmental and Molecular Toxicology, Oregon State University, Corvallis, Oregon 97331, USA

## Abstract

**Background:**

Regulatory decisions to phase-out the availability and use of common organophosphate pesticides among the general public were announced in 2000 and continued through 2004. Based on revised risk assessments, chlorpyrifos and diazinon were determined to pose unacceptable risks. To determine the impact of these decisions, organophosphate (OP) exposure incidents reported to the National Pesticide Information Center (NPIC) were analyzed for longitudinal trends.

**Methods:**

Non-occupational human exposure incidents reported to NPIC were grouped into pre- (1995–2000) and post-announcement periods (2001–2007). The number of total OP exposure incidents, as well as reports for chlorpyrifos, diazinon and malathion, were analyzed for significant differences between these two periods. The number of informational inquiries from the general public was analyzed over time as well.

**Results:**

The number of average annual OP-related exposure incidents reported to NPIC decreased significantly between the pre- and post-announcement periods (p < 0.001). A significant decrease in the number of chlorpyrifos and diazinon reports was observed over time (p < 0.001). No significant difference in the number of incident reports for malathion was observed (p = 0.4), which was not phased-out of residential use. Similar to exposure incidents, the number of informational inquiries received by NPIC declined over time following the phase-out announcement.

**Conclusion:**

Consistent with other findings, the number of chlorpyrifos and diazinon exposure incidents reported to NPIC significantly decreased following public announcement and targeted regulatory action.

## Background

In 1996, the Food Quality Protection Act (FQPA) was signed into law. This landmark legislation had a significant impact on the regulation of pesticides, addressing specific elements of exposure assessment and risk characterization. Among other changes, FQPA mandates additional protections for infants and children, considers aggregate and cumulative exposures and requires a periodic re-evaluation of pesticide registrations to include new scientific findings for each pesticide active ingredient.

**Figure 1:**
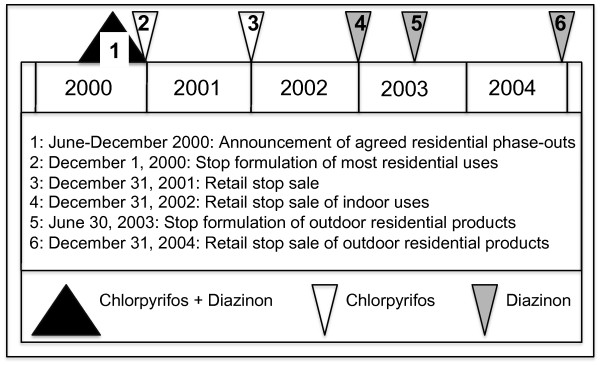
**Timeline for the phase-out of chlorpyrifos and diazinon residential use product availability**.

Organophosphates (OPs) were the first class of insecticides to have revised risk assessments under FQPA, in part because of a common mode of action shared among active ingredients. OPs elicit their effect via the phosphorylation and inhibition of acetylcholinesterase enzymes [1]. This leads to a build up of excess acetylcholine, a neurotransmitter essential to normal nerve impulse transmission. The risk assessments conducted for two common organophosphate insecticides, chlorpyrifos and diazinon, determined that inadequate protection existed for children based on current uses [2]. The risk mitigation decisions to phase out residential uses of chlorpyrifos and diazinon occurred from 2000 to 2004 (figure 1).

For chlorpyrifos, nearly all indoor and outdoor residential products were phased out. This risk mitigation decision gradually eliminated residential uses of chlorpyrifos, and targeted home lawn, indoor crack and crevice, and whole house termiticide treatments. Prior to this agreement, the Environmental Protection Agency (EPA) estimated that about 50% of all chlorpyrifos use was in and around the home [3]. For diazinon, all indoor residential and non-residential uses, as well as outdoor residential lawn and garden products, were cancelled from 2002–2004. It was estimated that diazinon represented 30% of the total homeowner use of insecticides prior to this agreement [4,5]. Malathion is another OP that is commonly used in numerous residential applications. In contrast to chlorpyrifos and diazinon, malathion was not phased out from residential use. The scope of the changes in the availability of chlorpyrifos and diazinon provides an opportunity to evaluate the effect of targeted regulatory actions on incident reporting.

One suitable source of data to evaluate trends in pesticide incidents is the National Pesticide Information Center (NPIC). NPIC is a cooperative agreement between Oregon State University and the Environmental Protection Agency that serves the United States, Puerto Rico and the Virgin Islands. One of the primary objectives of NPIC is to collect incident data related to pesticides. OP-related incidents reported to NPIC by the general public from 1995 through 2007 were analyzed prior to and following FQPA-initiated regulatory action. The objectives of this study are to determine if longitudinal differences exist in the number of reports for: 1) total OP incidents; 2) incidents among OPs targeted by regulatory action (chlorpyrifos and diazinon); and 3) incidents for a common residential OP not targeted by regulatory action (malathion). The trend in OP-related informational inquiries received by NPIC was examined over time as well.

## Methods

The data analyzed in this study were obtained from an incident database maintained by NPIC. The majority of incidents are reported over the telephone. Information is collected by a trained pesticide specialist, coded and archived in a secure database. For this study, an incident is defined as a person who was potentially exposed to a pesticide product by a known or plausible route of exposure, with or without symptoms. Furthermore, the event must have occurred less than two years ago and be reported by the affected party or someone with intimate knowledge of the event (i.e., family member or medical professional). The pubic can be referred to NPIC by multiple sources including: 1) the pesticide label of some products; 2) referrals from agencies, organizations and professionals; 3) the NPIC website and internet searches; and 4) outreach events.

Information that NPIC attempts to collect for all inquiries includes the active ingredient, EPA registration number and product name of the pesticide(s) of concern, reason for the call and location (state or zip code). For reported incidents, specialists collect information on signs and symptoms, exposure route(s), circumstances of exposure, and demographic characteristics such as gender and age. A medically trained toxicologist reviews all human incidents to determine the likelihood that reported symptoms and the exposure are consistent with the known toxicological and medical literature. All OP-related incidents that met the criteria described below were included for analysis. NPIC received 26,440 inquiries in 2008, including over 1500 human incident reports. Most of the reports to NPIC are from the general public.

Total OP incidents reported to NPIC for humans and animals were examined from 1995 to 2007. *A priori *criteria were developed for inclusion of incident reports to analyze in this study. The criteria were: 1) an OP was identified as the primary active ingredient reported in the incident and 2) the incident or exposure scenario was not occupational in nature. Occupational reports were excluded because the major focus of the announcement and regulatory action illustrated in figure 1 was for residential-use products and not agricultural, forestry, vector-control or other uses. In this study, residential use is defined as application inside the home or to the outdoor areas around a residence. A resident or pest control operator may perform the application.

Incident reports were grouped into two categories: a pre-announcement and post-announcement period. The pre-announcement period spanned from 1995 through the end of 2000. The post-announcement period began in 2001 and extended through 2007 for this study. Longitudinal trends in incident reports were examined for three OP active ingredients: chlorpyrifos, diazinon and malathion. Technically, the phase-out of diazinon occurred over several months following the agreement for chlorpyrifos (figure 1). However, public awareness and registrant action on diazinon products occurred at the same time as chlorpyrifos. Malathion is effectively a reference compound in this study since no regulatory action or agreement targeted this active ingredient during the time period of interest. In addition to the number of reports received by NPIC, OP-related incidents were classified by exposure route. The number of informational inquiries addressed by NPIC during the pre- and post-announcement periods was examined to determine potential changes over time.

OPs were searched in the NPIC database by their common active ingredient name. The active ingredients included were: acephate, azinphos-methyl, bensulide, bomyl, bromophos, bromophos-ethyl, cadusafos, carbophenothion, chlorethoxyfos, chlorfenvinphos, chlormephos, chlorphoxim, chlorpyrifos, chlorthiophos, coumaphos, crotoxyphos, crufomate, cyanofenphos, cythioate, DEF, demeton, demeton-S-methyl, dialifor, diazinon, dichlorofenthion, dichlorvos, dicrotophos, dimefos, dimethoate, dioxathion, disulfoton, ditalimfos, edifenphos, endothion, EPBP, EPN, ethion, ethoprop, ethyl parathion, etrimfos, famphur, fenamiphos, fenitrothion, fenophosphon, fensulfothion, fenthion, fonofos, formothion, fosthietan, feptenophos, hiometon, hosalone, IBP, iodofenphos, isazofos, isofenphos, isoxathion, leptophos, malathion, mephosfolan, merphos, methamidophos, methidathion, methyl parathion, methyl trithion, mevinphos, mipafox, monocrotophos, naled, oxydemeton-methyl, oxydeprofos, phencapton, phenthoate, phorate, phosalone, phosfolan, phosmet, phosphamidon, phostebupirim, phoxim, pirimiphos-ethyl, pirimiphos-methyl, phoxim, profenofos, poropetamphos, propyl thiopyrophosphate, prothoate, pyrazophos, pyridaphenthion, quinalphos, runnel, schradan, sulfotep, sulprofos, temephos, terbufos, tetrachlorvinphos, tetraethyl pyrophosphate, triazophos, and trichlorfon.

Data were analyzed using Levene's Test for Equal Variances, followed by an Independent Means Test, to determine whether there was a significant difference in incidents between pre- and post-announcement periods. These analyses were conducted for the average annual number of total OP incidents, and for chlorpyrifos, diazinon and malathion. Significance was defined as p ≤ 0.05. Statistical analyses were conducted with SPSS version 16.0. This study was approved by Oregon State University's Internal Review Board for use of de-identified data reported by the public to the National Pesticide Information Center.

## Results

**Figure 2:**
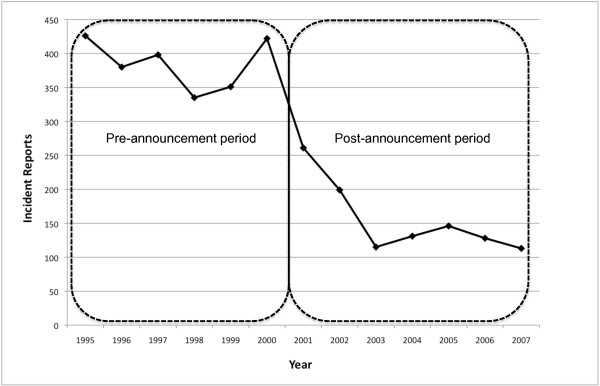
**Trend in total organophosphate incidents reported to NPIC during the pre- and post-announcement periods (p < 0.001)**.

NPIC received 3,385 OP-related incident reports involving a human exposure between 1995 and 2007 that met the criteria for inclusion in this study. Submitted reports ranged from a high of 426 incidents in 1995 to a low of 113 incidents reported in 2007. Chlorpyrifos, diazinon and malathion accounted for 85.4% of the total organophosphate exposure incident reports collected between 1995 and 2007. Of the total OP-related incidents in which exposure route was classified, 85.6% were due to dermal exposures. There was a statistically significant difference in the average annual number of OP incidents reported to NPIC between the pre- (1995–2000) and post-announcement (2001–2007) periods (p < 0.001) (figure 2). A steep decline in the number of incident reports was observed in 2001 and continued to drop sharply through 2003. To determine if the total number of incidents reported to NPIC increased or decreased over time, we examined all pesticide incidents from 1995–2007. In contrast to what was observed for OPs, no trend was observed for the total number of human exposure incidents reported to NPIC over time. The average annual number of incident reports was 1113 and 1204 in the pre- and post-announcement periods, respectively.

**Figure 3:**
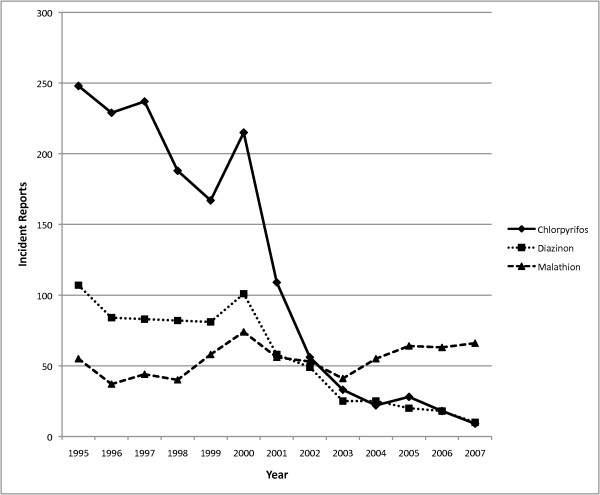
**Trends in chlorpyrifos, diazinon and malathion incidents reported to NPIC from 1995–2007**. A significant difference between pre- and post-announcement periods was observed for chlorpyrifos and diazinon (p < 0.001). No significant difference was observed for malathion (p = 0.4).

**Figure 4:**
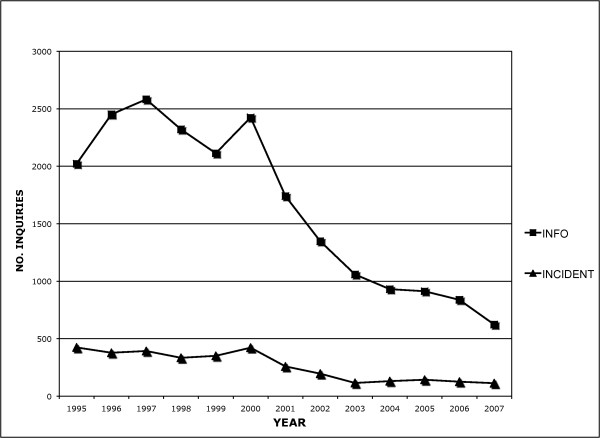
**Informational and incident inquiries involving organophosphates reported to NPIC from 1995–2007**.

Chlorpyrifos, diazinon and malathion incident reports were examined individually for differences in the number of reports received by NPIC during the pre- and post-announcement periods. Statistically significant differences in the annual average number of chlorpyrifos and diazinon incidents were observed between the pre- and post-announcement periods (p < 0.001). The mean annual number of reports for chlorpyrifos in the pre- and post-announcement phases was 214 and 39.3 incidents, respectively (Figure 3). Chlorpyrifos accounted for 55.5% of the OP-related incidents in the pre-announcement period, decreasing to 22.1% following regulatory action. For diazinon, an annual average of 80.6 reports were received pre-announcement compared with 19.6 incidents in the post-announcement period (Figure 3). In contrast, there was no significant difference in the number of malathion incidents between the pre- and post-announcement periods (p = 0.4). The average number of annual incidents was 51.3 and 56.9 in the pre- and post-announcement periods, respectively (Figure 3). Similar to the decline in incident reports received by NPIC, OP-related informational inquiries were substantially reduced between the two periods of interest (Figure 4). During the pre-announcement period, there was an annual average of 2319 inquires related to OPs. This declined to an average of 1065 annual inquires during the post-announcement period involving an OP. However, the percent of informational calls that were based on regulatory questions doubled from 4.3 to 8.6% following regulatory action.

## Discussion

The major finding in this study was a significant decrease in the number of OP-related incidents observed following the public announcement of residential phase-outs of chlorpyrifos and diazinon. This trend was driven by significantly fewer reports of diazinon and chlorpyrifos exposure incidents beginning in 2001 and continuing into 2003, coinciding with the changes in residential use and availability of these pesticides. In contrast, no significant trend in malathion incidents was observed over time. These findings are consistent with the observation that access to hazardous pesticides among the general public is an important predictor of poisonings and exposure incidents [6].

Poison Control Center data are valuable to examine trends in pesticide incidents among the general public as well. Sudakin and Power [7] analyzed the American Association of Poison Control Center, National Poison Data System (NPDS) database for longitudinal trends in OP-related incidents over time. Similar to our findings, a significant decrease in exposure incidents for total OPs was observed before FQPA-initiated regulatory action (1995–1999) compared with incidents reported from 2000–2004 [7]. In another study of NPDS data, reports of OP exposure incidents had decreased by 72% from 1995 to 2004 [8]. Data from the Washington Poison Control Center and Department of Health showed a rapid reduction in all OP cases, as well as chlorpyrifos and diazinon individually, following the phase-out announcement in the latter part of 2000 [9]. These studies support the trend in exposure incidents observed by NPIC.

The vast majority of exposure incidents received by NPIC come from the public and involve general use products. Residential use of pesticides is thought to be a major contributor to total pesticide exposure [10], especially when pest pressures are high [11]. Of the exposure incidents reported to NPIC, 85% were the result of unintentional dermal contact. These exposures occurred via multiple scenarios including spills, product malfunctions, misapplications, contact with wet, treated surfaces, and spraying into the wind. During the post-announcement period, NPIC received substantially fewer questions about OPs compared with the pre-announcement period. Questions from the public included issues related to toxicity and health, uncertainty about language on product labels, proper storage and disposal, and techniques to minimize exposure. Interestingly, the proportion of OP regulatory questions compared to all other OP-related inquiries doubled following the restriction of chlorpyrifos and diazinon availability, suggesting interest among the public following the phase-out announcement.

Chlorpyrifos and diazinon accounted for 56% and 23%, respectively, of all OP-related incidents reported to NPIC during the pre-announcement period. This is not surprising given the number of products that contained these active ingredients. Chlorpyrifos, first registered in 1965, had numerous applications for homes, lawns and pets. Common product names included Durban, Lorsban, Suscon, Radar, Killmaster, Empire, with over 400 commercial products available at one point [12]. Formulations of chlorpyrifos included gels, emulsifiable concentrates, granular, wettable powders, and microencapsulated products. Today, chlorpyrifos is mostly used on corn, soybeans and tree nuts and fruits in U.S. agriculture. Diazinon was first registered in 1956 in the United States. It was found in a variety of products and formulations including Spectracide, Bonide, Dragon, Re-Zist and Spray Kill. During the post-announcement period (2001–2007), chlorpyrifos and diazinon accounted for only 22 and 18% of total OP-related incidents, respectively, presumably as a result of the FQPA-initiated regulatory actions.

Relative to other organophosphates, malathion accounted for 39.5% of all incidents reported to NPIC during the post-announcement period, a three-fold increase compared with the pre-announcement period. The increase in the percent of OP incidents attributable to malathion is likely the result of the actions taken for diazinon and chlorpyrifos. Recently, EPA and malathion registrants agreed to terminate uses of malathion in indoor premises and hard surfaces, residential dust and pressurized can formulations, as well as broadcast applications for residential lawns, effective December 3, 2008 [13]. Based on our analysis of diazinon and chlorpyrifos, we anticipate fewer malathion exposure incidents reported by the general public.

The primary limitation of our study is the self-reported nature of the data received by NPIC. While NPIC specialists are trained to collect detailed information about the symptoms, circumstances surrounding exposures and detailed product information, several issues may compromise this ability. These include the potential for the public to provide inaccurate or incomplete information, recall bias and the inability to capture relevant information for incidents that require an immediate transfer to a poison control center. It should be noted that emergent cases transferred to Poison Control Centers, in which an OP was identified, were included for analysis in this study. Awareness of NPIC services among the public and among officials in a position to refer individuals to our Center may vary geographically and over time. This could influence the frequency of reports submitted to NPIC for any given year.

## Conclusion

These findings suggest that FQPA-risk mitigation decisions for chlorpyrifos and diazinon were associated with a significant decline in the number of human exposure incidents involving these insecticides, based on reports collected by NPIC. These findings are consistent with the results of other studies that also suggest regulatory changes were associated with a significant decline in the number of OP incidents prior to and following the announcement that gradually eliminated public availability of these products.

## Abbreviations

EPA: Environmental Protection Agency; FQPA: Food Quality Protection Act; NPIC: National Pesticide Information Center; OP: organophosphate; NPDS: National Poison Data System.

## Competing interests

The authors declare that they have no competing interests.

## Authors' contributions

All authors are principal investigators of NPIC. DLS is the director of NPIC and carried out the analysis of the data and drafted the manuscript. DLS and JJJ assisted with interpretation of results and reviewed the manuscript. All authors read and approved the final manuscript.
